# Impact of Processing Parameters on Ti Schottky Contacts on 4H-SiC

**DOI:** 10.3390/ma18071447

**Published:** 2025-03-25

**Authors:** Marilena Vivona, Gabriele Bellocchi, Valeria Puglisi, Corrado Bongiorno, Salvatore Adamo, Filippo Giannazzo, Simone Rascunà, Fabrizio Roccaforte

**Affiliations:** 1 Consiglio Nazionale delle Ricerche, Istituto per la Microelettronica e Microsistemi (CNR-IMM), Strada VIII, n. 5—Zona Industriale, 95121 Catania, Italy; corrado.bongiorno@imm.cnr.it (C.B.); filippo.giannazzo@imm.cnr.it (F.G.); fabrizio.roccaforte@imm.cnr.it (F.R.); 2 STMicroelectronics, Stradale Primosole 50, 95121 Catania, Italy; gabriele.bellocchi@st.com (G.B.); valeria-sst.puglisi@st.com (V.P.); salvatore.adamo1@st.com (S.A.); simone.rascuna@st.com (S.R.)

**Keywords:** 4H-SiC, Schottky barrier, Titanium, Schottky contact processing, electrical characterization

## Abstract

In this paper, we investigated the effects of the processing parameters, such as deposition methods, annealing temperature, and metal thickness, on the electrical characteristics of Ti/4H-SiC contacts. A reduction of the Schottky barrier height from 1.19 to 1.00 eV following an increase of the annealing temperature (475–700 °C) was observed for a reference contact with an 80 nm-thick Ti layer. The current transport mechanisms can be described according to the thermionic emission (TE) and thermionic field emission (TFE) models under forward and reverse biases, respectively. The comparison with an e-beam evaporated Ti(80 nm)/4H-SiC contact did not show significant differences for the forward characteristics, while an increase of the leakage current was observed under high reverse voltage (>500 V). Finally, a thickness variation from 10 to 80 nm induced a reduction of the Schottky barrier height, due to the reaction occurring at the interface with a Ti-Al region extended up to the 4H-SiC surface. In addition to a deeper understanding of the Schottky barrier properties, this work is useful for the development of Schottky barrier diodes with tailored characteristics.

## 1. Introduction

Today, the hexagonal polytype of silicon carbide (4H-SiC) is the most promising wide band gap semiconductor for the development of efficient power electronics devices [1]. In particular, the superior performance of 4H-SiC devices with respect to the traditional silicon (Si) ones arises from the outstanding electronic properties of the material, e.g., a wide bandgap (3.26 eV), high critical electrical field (>2 MV/cm), saturated drift velocity (>2 × 10^7^ cm × s^−1^), and high thermal conductivity (4.9 W cm^−1^ K^−1^) [2]. In addition, the crystal quality and wafer size of the commercially available 4H-SiC material have steadily improved over the years, thus leading to the worldwide establishment of several 200 mm 4H-SiC device production fabs [3,4].

Currently, the metal-oxide-semiconductor field-effect transistors (MOSFETs) and Schottky barrier diodes (SBDs) are the most mature 4H-SiC devices on the technological level and are largely employed in several applications. In particular, the ideal choice for using 4H-SiC devices falls for operations in the medium- and high-voltage range (600–1700 V) [5], spacing from automotive, industrial motors up to transportation and power grids. In spite of the huge progresses recorded in 4H-SiC technology, the fine optimization of some crucial device processing steps is always among the objectives of both the academic and industrial worlds. For instance, one of the main concerns in 4H-SiC SBD technology is the control and full understanding of the electrical properties of metal/4H-SiC Schottky barrier height (*Φ_B_*) [6]. In fact, the precise control of the Schottky barrier properties is mandatory for achieving reproducible performance with tailored characteristics and more efficient electronic operations of the diodes [7].

In the last decades, several studies have investigated the electrical properties of a variety of metallization schemes for Schottky contacts to 4H-SiC and the current transport through these interfaces [6,8,9,10,11]. In fact, the choice of appropriate metal is related to the envisaged application. For instance, Ni is preferred for sensing or detection applications [12], as Ni-silicides featuring high Schottky barrier height (around 1.60 eV), which favor low level of leakage current. The high work-function of Ni also allows the effect of surface treatment on the Schottky barrier to be investigated [13]. On the other hand, low-work function metals, such as Mo [14,15,16,17], W [15,18,19,20], and Ti [21,22], have recently emerged thanks to the possibility to form low Schottky barriers, which entail a minimization of the diode power consumption [7]. Among them, Ti contacts are currently a well-established industrial solution for device manufacturing, offering a high reproducibility with barrier height values ranging between 0.78 and 1.33 eV for as-deposited and annealed contacts [7,22,23], with the *Φ_B_* tendentially higher after annealing of the Ti/4H-SiC contact. A survey of the Schottky barrier height derived from literature on annealed Ti/4H-SiC contacts is reported in Table 1.

**Figure 1:**
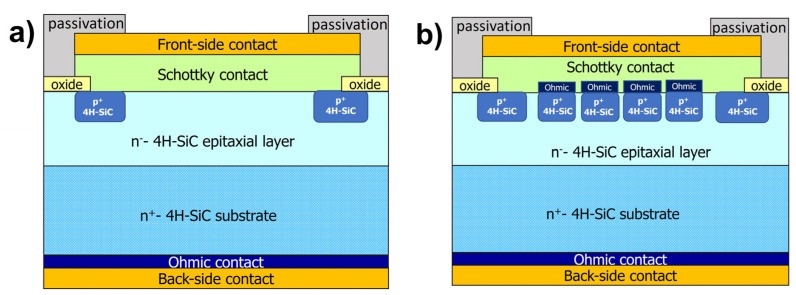
Cross-section schemes of (**a**) 4H-SiC Schottky barrier diode (SBD) and (**b**) junction-Schottky barrier (JBS) diode.

However, an improved control on the *Φ_B_* variability range can be achieved by well-defined processing and an accurate evaluation of all the parameters involved in the contact fabrication. Additionally, performance improvement can be obtained also by operating on the device layout. For instance, the so-called junction barrier Schottky (JBS) diode is widely considered for the high level of rectifying performance. In practice, the JBS layout combines the advantage of the low forward voltage drop of a conventional Schottky diode with the hard breakdown and low leakage of a p-n junction system [28], thanks to the presence of p^+^-type regions embedded in an n-type epitaxial area [29,30]. The general schemes of an SBD and a JBS diode are depicted in Figure 1a and Figure 1b, respectively.

As for conventional SBDs, the core of JBS devices is the metal/4H-SiC interface, whose properties entail the electrical behavior of the entire device, and the optimization of this part is at the base for further development of SBD technology.

In this paper, we investigated the impact of some processing parameters (i.e., annealing temperature, deposition method and thickness) on the electrical characteristics of Ti Schottky contacts to 4H-SiC. The electrical behavior of the diodes was characterized under forward and reverse bias and correlated to a microstructural analysis of the metal/4H-SiC interface performed by transmission electron microscopy (TEM). In particular, the independence of the deposition methods (e-beam or sputtering) was highlighted for Ti/4H-SiC contact, whereas the temperature of the annealing treatment and the thickness of the film affect the Schottky barrier height of the diode.

## 2. Experimental

The material of our study was a “production grade” 9.5-μm thick 4H-SiC epitaxial layer, intentionally doped with nitrogen (n-type doping concentration of N_D_ = 8 × 10^15^ cm^−3^), grown onto a heavily doped 4H-SiC (0001) substrate. On this sample, Schottky diodes with an area of 3.35 mm^2^ were fabricated. Ti was used as barrier metal and Schottky contacts of different thickness were defined on the front-side of the sample by optical lithography and lift-off processing steps. In particular, thick (80 nm) Ti contacts were deposited by DC magnetron sputtering, while e-beam evaporation was used for thinner Ti layers (ranging from 10 to 50 nm). A reference sample of 80-nm thick Ti/4H-SiC was also fabricated by e-beam evaporation for comparison with the sputtering deposition method. In order to prevent Ti oxidation when moving from the evaporation chamber to the sputtering chamber for the deposition of the final thick metal layer (an AlSiCu alloy), a few nanometers thin Al layer was evaporated on the Ti Schottky barrier layer. The contacts were then subjected to 10-min thermal annealing treatments in N_2_ atmosphere, at temperatures ranging from 475 °C to 800 °C. The electrical characterization of the contacts was performed by means of current–voltage (I–V) measurements in a Karl–Suss MicroTec probe station equipped with a parameter analyzer (B1505 A by Keysight Technologies, Santa Rosa, CA, USA) enabling to detect current levels in the order of pA. The electrical parameters featuring the contacts (ideality factor n and Schottky barrier height *Φ_B_*) were averaged over a set of measurements on a set of 20 equivalent diodes. Furthermore, the microscopic modifications that occurred at the metal/semiconductor interface were monitored through transmission electron microscopy (TEM) in cross-section using a 200 kV 2010F microscope by JEOL (Tokyo, Japan).

## 3. Results and Discussion

**Figure 2:**
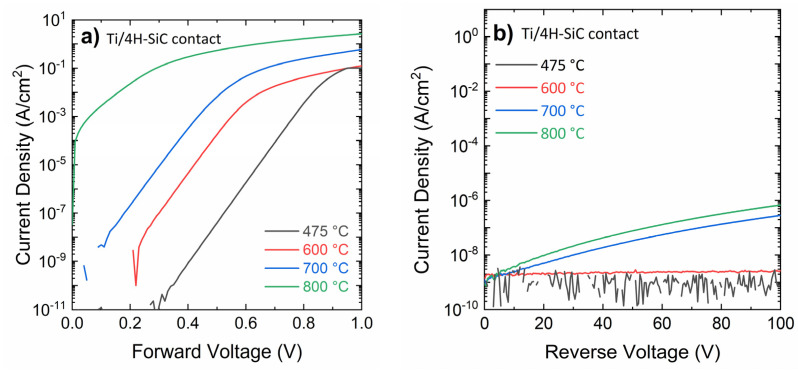
(**a**) Forward and (**b**) reverse electrical characteristics of Schottky diodes with 80 nm-Ti Schottky contact, subjected to thermal annealing at various temperatures (475 °C, 600 °C, 700 °C, and 800 °C).

Firstly, we characterized the devices fabricated with 80-nm-thick sputtered Ti Schottky barrier metal, and subjected to thermal annealing treatments at 475 °C, 600 °C, 700 °C and 800 °C. Here, the 475 °C is considered as the reference, since this process is adopted to stabilize the thick AlSiCu metal on the top of the Schottky metal. The forward and reverse current density-voltage (J-V) characteristics of these diodes are reported in a semilog scale in Figure 2a and Figure 2b, respectively.

As can be observed in Figure 2a, up to the annealing temperature of 700 °C, the forward J-V characteristics of the Ti/4H-SiC diodes showed a wide linear region in a semilog scale, extending over 6–7 orders of magnitudes. However, a gradual shift of the forward characteristics towards lower turn-on voltages was observed with increasing annealing temperature. After annealing at 800 °C, the diode turn-on and the linear region were notably reduced.

When the diodes were polarized under reverse bias (Figure 2b), the leakage current increased from the noise level (reference 475 °C annealed contact) up to 10^−6^ A/cm^2^ at 100 V for the 800 °C annealed contact.

For a better understanding of the electrical behavior of the Schottky diodes, we studied the current transport mechanisms at the interface under both forward and reverse bias. In particular, the ideality factor n and Schottky barrier height *Φ_B_* were derived, applying the thermionic emission (TE) model when fitting the linear region in the semilog plot of the J-V curves [31]:

(1) $$J_{F}=A^{*}T^{2}exp\left[\left(-\frac{q\Phi_{B}}{k_{B}T}\right)\right]exp\left[\left(\frac{qV_{F}}{{nk}_{B}T}\right)\right]=1,$$

where A* is the effective Richardson constant of 4H-SiC (146 A × cm^−2^ × K^−2^) [10], *T* is the absolute temperature, *q* is the elementary charge, *k_B_* is the Boltzmann constant, and *V_F_* is the applied forward voltage.

**Figure 3:**
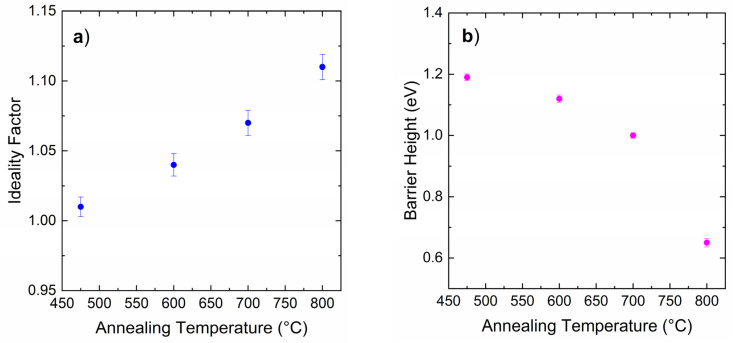
Ideality factor (**a**) and Schottky barrier height (**b**) values derived by applying the TE model to the forward characteristics of the Ti/4H-SiC Schottky diodes subjected to thermal annealing treatment at temperatures of 475 °C, 600 °C, 700 °C, and 800 °C.

The values of n and *Φ_B_* extracted at each annealing temperature are reported in Figure 3a and Figure 3b, respectively. An almost ideal behavior was observed, with n values remaining below 1.10 after the annealing at of 475 °C, 600 °C, and 700 °C. Instead, the thermal treatment at 800 °C degraded the electrical characteristic for the contact, with a reduced linear region that made the extrapolation of the ideality factor and Schottky barrier height values more difficult. For the Ti/4H-SiC Schottky diodes presenting an extended linear region, i.e., those annealed in the range 475–700 °C, the barrier height *Φ_B_* decreased from 1.19 eV to 1.00 eV with increasing annealing temperature. This effectively tuned the Schottky barrier height in a range of 190 meV by varying the annealing temperature from 475 °C to 700 °C.

Under reverse bias, the leakage current of the diodes increased with increasing annealing temperature, with only the 700 °C and 800 °C annealed samples presenting leakage current above the sensitivity limit of our experimental set-up. Even when a degradation of the forward characteristic of the 800 °C annealed contact was observed, the leakage current remained on a level similar to the of the 700 °C annealed contact.

Here, the current transport under reverse bias was explained by taking into account a tunneling contribution to the current, according to the thermionic field emission (TFE) theory [32], using the following relation between reverse current density *J_R_* and voltage *V_R_* [33]:

(2) $$J_{R}=A^{*}T^{2}\sqrt{\frac{{q\pi E}_{00}}{kT}}\sqrt{V_{R}+\frac{\Phi_{B}}{cosh{\left(\frac{qE_{00}}{kT}\right)}^{2}}}\;exp\left(-\frac{\Phi_{B}}{E_{1}}\right)exp\left(\frac{V_{R}}{E_{2}}\right)$$

with *h* the Planck constant, m* the effective mass of electron and *εSiC* the dielectric constant of the semiconductor. The *E*_00_, *E*_1_ and *E*_2_ functions are expressed as $E_{00}=(h/4\pi )\sqrt{N_{D}/m^{*}\varepsilon_{SiC}}$, ${E_{1}=E}_{00}\times \tanh{\left(qE_{00}/kT\right)}^{-1}$ and $E_{2}=E_{00}\times {\left(qE_{00}/kT-tanh\left(qE_{00}/kT\right)\right)}^{-1}$, where an effective mass of 0.39 × m_0_ and a dielectric constant of 9.76 × ε_0_ were used (with m_0_ the free electron mass and ε_0_ the vacuum permittivity).

**Figure 4:**
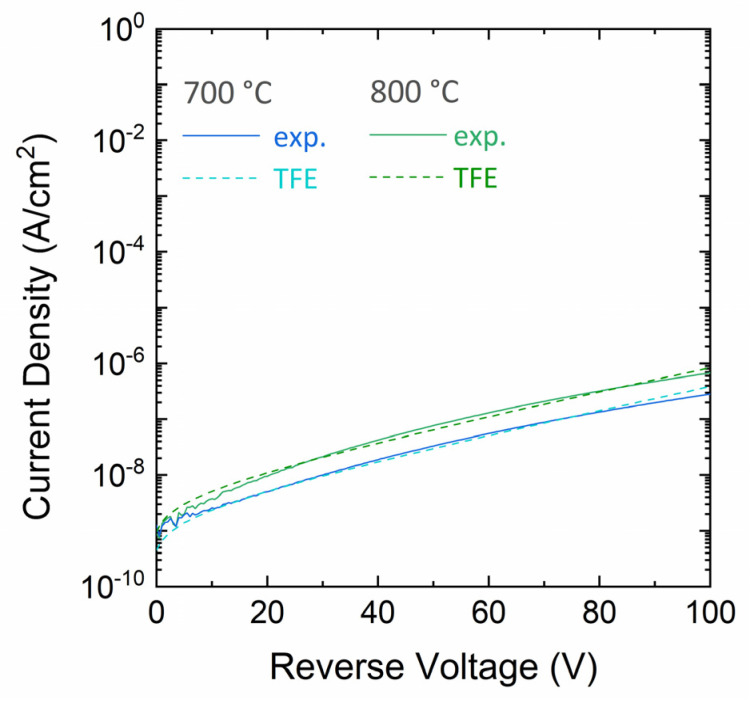
Experimental and simulated (TFE model) reverse current density-voltage (J-V) characteristics of the Ti/4H-SiC Schottky diodes annealed at 700 °C and 800 °C.

For the 700 °C and 800 °C annealed Ti/4H-SiC contacts, for which the current level was well above the sensitivity limit, the experimental and fitted reverse curves according to the TFE theory are reported in Figure 4.

For the Schottky barrier height, the *Φ_B_* values were derived from the fit of the experimental curves, according to the TFE model. Specifically, *Φ_B_* was 1.00 eV and 0.98 eV for the 700 °C and 800 °C–annealed Ti/4H-SiC contact, respectively. Then, the effect of the metal deposition technique on the electrical properties of the Ti/4H-SiC contact was also monitored. Specifically, the different mechanisms associated to metal deposition methods of our study, i.e., evaporation and sputtering, can affect the physical properties of the film and, ultimately, the electrical characteristics of the diodes.

**Figure 5:**
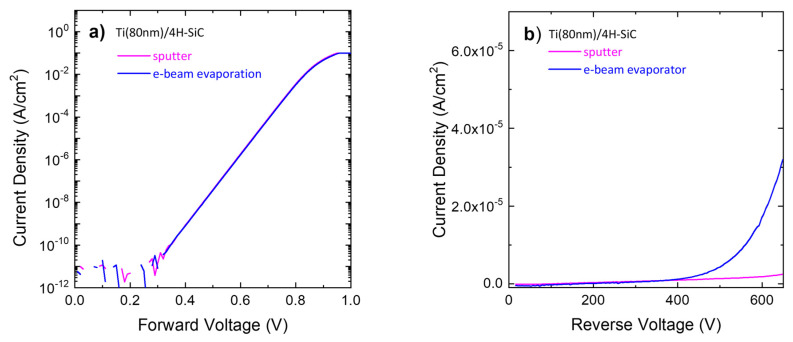
(**a**) Forward and (**b**) reverse electrical characteristics of 475 °C annealed Ti (80 nm)/4H-SiC contact deposited by sputtering or e-beam evaporation.

To this aim, we compared the forward and reverse electrical behavior of the lowest temperature annealed Ti(80 nm)/4H-SiC Schottky diode fabricated by the two different deposition techniques. The forward and reverse electrical characteristics are reported in Figure 5a and Figure 5b, respectively.

The forward characteristics (Figure 5a) demonstrated an independence of the deposition method for the two contacts. Instead, under reverse bias (Figure 5b), the two contacts featured similar characteristics up to about 400 V, while above a sudden current increase was observed for the evaporated Ti/4H-SiC contact.

**Figure 6:**
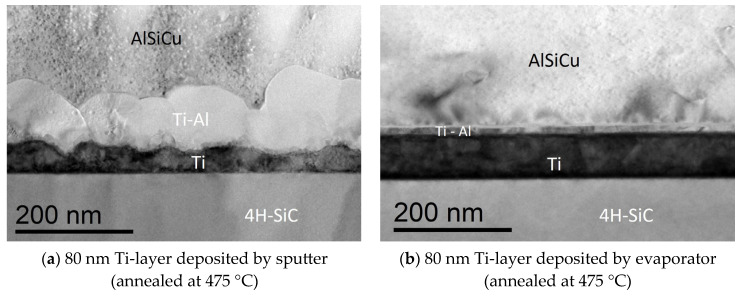
Cross-section TEM micrographs related to the Ti/4H-SiC interface annealed at 475 °C for (**a**) sputtered and (**b**) evaporated 80 nm-Ti layer.

To better understand the Ti/4H-SiC interface formation, a microstructural analysis was carried out by TEM in cross-section on the two 80 nm Ti samples annealed at 475 °C, deposited either by sputtering (Figure 6a) or e-beam evaporation (Figure 6b).

In the Ti-sputtered/4H-SiC contact, a reaction between Ti and Al was observed in the upper part of the contact, with the Al coming from the AlSiCu protective layer. This reaction produced the Ti-Al grains, according to the solid-state reactions occurring in Ti/Al bilayers subjected to thermal annealing processes [34]. Additionally, we observed that only Ti was directly in contact with the 4H-SiC surface. Also, for Ti-layer deposited by evaporation, a Ti-Al reaction appeared, in this case probably due to the presence of the additional Al film evaporated sequentially to the Ti film with the aim to avoid the exposure to air of the Ti surface before AlSiCu layer sputtering. This Ti-Al region was very confined in the few tens of nanometers of the upper part of the contact, with the interfacial region between Ti and 4H-SiC similar in the two cases, i.e., a layer of unreacted Ti in contact to 4H-SiC. This can explain the origin of the same electrical characteristics observed under forward bias and under reverse bias up to 400 V in the sputtered and evaporated Ti/4H-SiC contacts.

**Figure 7:**
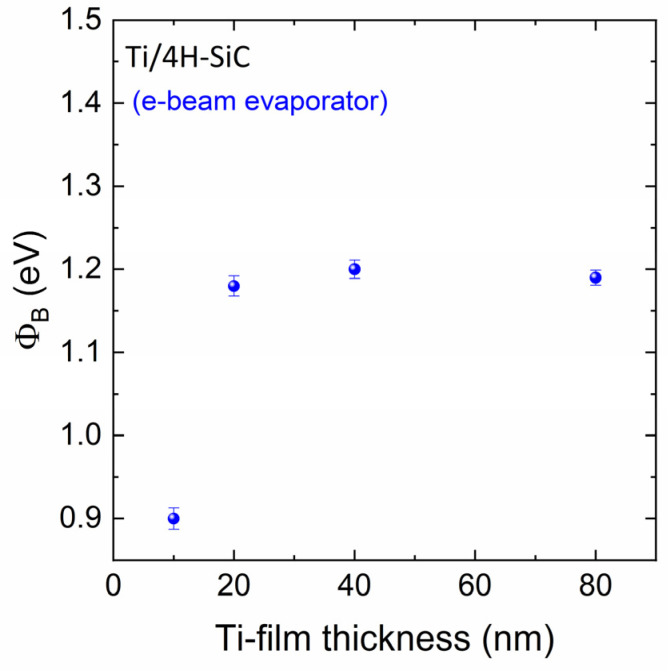
Barrier height values extrapolated from the electrical characteristics of e-beam evaporated Ti-layer on 4H-SiC with a thickness of 10, 20, 40, and 80 nm.

Finally, we considered the effect of the Ti-film thickness on the Schottky barrier height of the contact. The Ti-layer from 80 nm down to 10 nm-thick Ti film, fabricated by the e-beam evaporation technique, which allowed good control for a thinner layer, was investigated. The *Φ_B_* values, extrapolated from the forward characteristics, varying from 0.9 eV for the 10 nm-thick Ti film, increased to about 1.20 eV for the 20, 40 nm, and 80 nm Ti contact, as shown in Figure 7.

**Figure 8:**
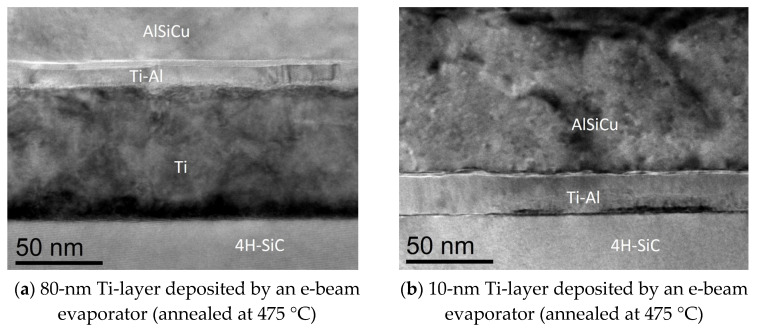
(**a**,**b**) Cross-section TEM analyses related to the Ti/4H-SiC interface annealed at 475 °C for evaporated Ti-layer with a thickness of 80 nm and 10 nm, respectively.

The Ti-film thickness reduction was also considered in relation to a possible interaction of the AlSiCu layer directly with the 4H-SiC. In fact, the barrier lowering with metal layer thickness thinning could be related to the reactions occurred at the interface during the annealing treatments in processing. The microstructural analyses, performed by TEM analysis on the thickest and thinnest samples reported in Figure 8a (80-nm-thick Ti-layer) and Figure 8b (10-nm-thick Ti-layer), revealed that the reacted region Ti-Al extended up to the 4H-SiC surface for the thinnest Ti-layer (Figure 8b). This can be considered at the base of the barrier height reduction in this case.

Obviously, different reactions can occur in the contact region with higher annealing temperature (≥1000 °C), as discussed in previous papers [35,36,37], which detected the presence of a ternary phase (Ti_3_SiC_2_) by means of X-ray photoelectron spectroscopy (XPS) or X-ray diffraction (XRD) analysis in contact with Ohmic characteristics.

The Ti/4H-SiC contacts characterized in this work present very good Schottky electrical behavior (extended linear region under forward bias and low level of the leakage current). With an appropriate choice of the processing conditions (annealing temperature, deposition method and thickness), it is possible to obtain accurate control of the Schottky barrier height over hundreds of meV). This work clearly demonstrates the relation between the Schottky barrier height value and the processing condition. Particularly, this kind of Ti-based contacts can be used in various applications, spacing from power electronics devices [23] to high-resolution alpha spectroscopy detectors [38].

Additional characterizations for studying the inhomogeneity at nanometric scale in the metal/semiconductor interface for instance based on fractional model [39,40] or the common adopted approach based on conventional current-voltage-temperature study [41,42] could be useful to highlight the effect of the local structure of the contact on the current transport mechanisms.

## 4. Conclusions

In this paper, the role of the processing parameters is evaluated in controlling the electrical characteristics of the barrier in Ti/4H-SiC Schottky. Specifically, the annealing temperature, deposition method, and metal thickness were studied.

Regarding the temperature of the annealing treatment, a reduction of the Schottky barrier height was obtained following an increase of the annealing temperature for reference contact with an 80-nm-thick Ti layer. Specifically, the barrier height varied between 1.00 and 1.19 eV, with the ideality factor lower than 1.10. The current transport mechanisms can be described according to the TE and TFE models under forward and reverse biases, respectively. The comparison with an e-beam evaporated Ti (80 nm)/4H-SiC contact did not show differences for the forward characteristics, while an increase in the leakage current was observed under high reverse voltage (>500 V). The presence of an Al-film deposited to limit the air exposure of Ti film in the processing of Ti/4H-SiC evaporated contacts limited the formation of the Ti-Al phase (as observed in 80-nm-thick sputtered Ti contact) in only a few tens of nanometers of the upper part of the contact, while an unreacted Ti-layer remained in contact with the 4H-SiC surface. This can explain the similar behavior under forward bias in the two cases. Finally, a thickness variation from 10 to 80 nm induced variation in the Schottky barrier height, with the reacted Ti-Al region able to arrive in the 4H-SiC surface.

Besides a deeper understanding of the Schottky barrier properties, this study also provides useful insights for device manufacturers to optimize the diode’s layout and obtain the desired characteristics.

## Figures and Tables

**Table 1:** Survey of the Schottky barrier height derived from the Ti/n-type 4H-SiC Schottky contacts literature.

Metal (Thickness)	Contact Fabrication Conditions	*Φ_B_*	Reference
Ti (n.r.)	e-beam evaporation, annealing at 500 °C and 600 °C; for 10 min in Ar	1.15–1.22 eV	[24]
Ti (200 nm)	Sputter deposition with substrate T ranging from 28 °C to 500 °C; annealing for 60 h at 500 °C in vacuum	0.83–1.13 eV	[25]
Ti (100 nm)	e-gun evaporation, annealing at 500 °C and 750 °C for 2 min in N_2_	1.23–1.33 eV	[26]
Ti (100 nm)	Deposition, annealing at 450 °C and 650 °C for 0.5 and 1 h in vacuum	1.27 eV	[27]

n.r. = not reported.

## Data Availability

The original contributions presented in this study are included in the article. Further inquiries can be directed to the corresponding author.
